# *Collinsella aerofaciens* as a predictive marker of response to probiotic treatment in non-constipated irritable bowel syndrome

**DOI:** 10.1080/19490976.2023.2298246

**Published:** 2024-01-04

**Authors:** Giorgio Gargari, Giacomo Mantegazza, Cesare Cremon, Valentina Taverniti, Alice Valenza, Maria Raffaella Barbaro, Giovanni Marasco, Robin Duncan, Walter Fiore, Roberto Ferrari, Valerio De Vitis, Giovanni Barbara, Simone Guglielmetti

**Affiliations:** a Division of Food Microbiology and Bioprocesses, Department of Food, Environmental and Nutritional Sciences (DeFENS), University of Milan, Milan, Italy; b Dipartimento di Scienze Mediche e Chirurgiche, IRCCS Azienda Ospedaliero-Universitaria di Bologna, Bologna, Italy; c Department of Medical and Surgical Sciences, University of Bologna, Bologna, Italy; d Sofar SpA, Trezzano Rosa, Italy

**Keywords:** Dorea, Coriobacteriaceae, 16S rRNA gene profiling, PV-1, diarrhea-predominant IBS, mixed-type IBS, dysbiotic-type IBS, gut-liver axis

## Abstract

Probiotics are exploited for adjuvant treatment in IBS, but reliable guidance for selecting the appropriate probiotic to adopt for different forms of IBS is lacking. We aimed to identify markers for recognizing non-constipated (NC) IBS patients that may show significant clinical improvements upon treatment with the probiotic strain *Lacticaseibacillus paracasei* DG (LDG). To this purpose, we performed a post-hoc analysis of samples collected during a multicenter, double-blind, parallel-group, placebo-controlled trial in which NC-IBS patients were randomized to receive at least 24 billion CFU LDG or placebo capsules *b.i.d*. for 12 weeks. The primary clinical endpoint was the composite response based on improved abdominal pain and fecal type. The fecal microbiome and serum markers of intestinal (PV1 and zonulin), liver, and kidney functions were investigated. We found that responders (R) in the probiotic arm (25%) differed from non-responders (NR) based on the abundance of 18 bacterial taxa, including the families *Coriobacteriaceae*, *Dorea* spp. and *Collinsella aerofaciens*, which were overrepresented in R patients. These taxa also distinguished R (but not NR) patients from healthy controls. Probiotic intervention significantly reduced the abundance of these bacteria in R, but not in NR. Analogous results emerged for *C. aerofaciens* from the analysis of data from a previous trial on IBS with the same probiotic. Finally, *C. aerofaciens* was positively correlated with the plasmalemmal vesicle associated protein-1 (PV-1) and the markers of liver function. In conclusion, LDG is effective on NC-IBS patients with NC-IBS with a greater abundance of potential pathobionts. Among these, *C. aerofaciens* has emerged as a potential predictor of probiotic efficacy.

## GRAPHICAL ABSTRACT

**Figure:**
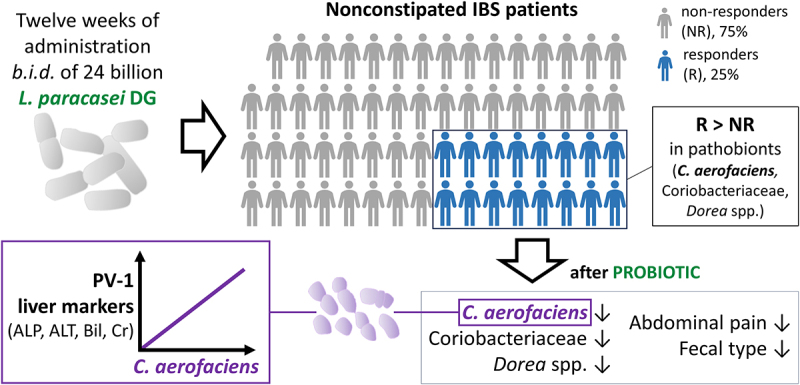

## Introduction

Irritable bowel syndrome (IBS) is a common disorder of gut-brain interaction in which recurrent abdominal pain is associated with defecation or a change in bowel habits.^1^ IBS is a complex and multifactorial condition that may be associated with several potential factors and mechanisms, such as altered intestinal serotonin level and metabolism,^2,3^ decreased density of peptide YY (PYY) cells in the colon,^4,5^ high levels of histamine and elevated number of mast cells in intestinal mucosa,^6^ low-grade mucosal inflammation,^7^ compromised epithelial barrier^8^ and altered gut microbiome,^9^ which can lead to low-grade mucosal inflammation with visceral hypersensitivity.^7^ However, these aspects can variably and not simultaneously contribute to the symptomatology of IBS patients, which could be hypothetically stratified into a wider number of IBS forms that are currently unknown. According to Rome IV criteria, IBS is subdivided into categories according to bowel habits, including IBS with predominant constipation (IBS-C), IBS with predominant diarrhea (IBS-D), and IBS with mixed bowel habit (IBS-M).^10^ Ongoing research aims to unravel the complexities of IBS behind this classification and develop targeted therapies to address these underlying mechanisms.

Various therapeutic options for IBS target the underlying pathophysiological aspects of the condition. Unfortunately, no single approach can effectively address this disorder’s diverse manifestations simultaneously. The most common approaches include anti-pain medications (e.g., antispasmodics and neuromodulators), dietary pattern modification (e.g., low-FODMAP diet), psychological intervention, antibiotics (e.g., rifaximin), opioid receptor agonists (e.g., eluxadoline), antagonists of serotonin receptors (e.g., alosetron, ondansetron, ramosetron), secretagogues (e.g., linaclotide), and other medications. In addition, since there is a growing body of evidence indicating the implication of altered intestinal microbiota in IBS,^11^ supplementation with probiotics and/or prebiotics is often exploited as adjuvant treatment.^12,13^

Although specific microbial strains have shown promising results,^14^ meta-analyses have reported inconsistent findings concerning the efficacy of probiotics for various IBS outcome measures, potentially due to wide variability among the probiotic formulations considered [differences in microbial strain(s), dosage, and administration protocol] and pathophysiological differences in IBS subtypes^15^. Reliable guidelines for selecting appropriate probiotic strains and/or formulations for treating the different forms of IBS are currently lacking.

*Lacticaseibacillus paracasei* DG is a probiotic bacterium experimentally proven to survive gastrointestinal transit in adults^16^ and children,^17^ to modify the composition of the intestinal microbial ecosystem in healthy adults,^18^ and to modulate immune responses in different intestinal conditions.^19^ In addition, a multicenter, randomized, double-blind, crossover, placebo-controlled pilot trial (PROBE-IBS/1; ClinicalTrials.gov Identifier: NCT02371499) showed that strain DG (at least 24 billion CFU/capsule, two capsules per day) induced a significant reduction in *Ruminococcus* spp., a significant increase in fecal acetate and butyrate, and a significant reduction in the pro-inflammatory cytokine interleukin-15 when administered to IBS patients.^20^ The results of the PROBE-IBS/1 trial suggested that the clinical efficacy of *L. paracasei* DG could be greater in patients with IBS-D and IBS-M and encouraged the conduct of a larger study (named PROBE-IBS/2) to assess the effect of Enterolactis^Ⓡ^ PLUS capsules (a single-strain probiotic formulation containing at least 24 billion CFUs of *L. paracasei* DG) on abdominal symptoms in non-constipated IBS (NC-IBS) patients without constipation. Here, we present the results of a post-hoc analysis of fecal and serum samples collected from NC-IBS patients in the probiotic arm during the PROBE-IBS/2 study with the aim of identifying potential markers distinguishing the population of patients who achieved the primary clinical endpoint of the trial (responders; R) from non-responders (NR). These results suggest that R patients are characterized by an increased abundance of potential pathobionts, which can be mechanistically linked to the onset of IBS symptoms and can be reduced by intake of the probiotic bacterium *L. paracasei* DG.

## Results

### Characteristics of non-constipated IBS patients according to clinical responsiveness

According to the primary endpoint of the PROBE-IBS/2 trial (i.e., composite response over 12 weeks), 16 patients with NC-IBS were considered responders (R: 25.4%). At baseline, R patients were not significantly different from non-responders (NR) patients (*n* = 47; 74.6%) in terms of age, abdominal pain, fecal type, fecal organic acids, serum markers for intestinal permeability and functioning [i.e., citrulline, plasmalemmal vesicle associated protein-1 (PV-1), and zonulin], and liver and kidney function blood markers (Table 1). In contrast, the mean abdominal pain calculated during 12 weeks of treatment was significantly lower in the R group than in the NR group. As expected, abdominal pain and fecal type calculated during the 12-week probiotic intervention period were significantly lower than those during the 2-week run-in (baseline) period only in the R group. In contrast, probiotic treatment did not significantly affect the concentrations of organic acids in feces and serum markers (Table 1).

**Table 1.** Characteristics of the non-constipated IBS patients considered in this study. Mean values ± standard deviation for each parameter are reported.

		Total (*n* = 63; 100%)	Responders (*n* = 16; 25.4%)	Non-responders (*n* = 47; 74.6%)
Age (years)		35 ± 12	33 ± 11	35 ± 13
Female sex [n (percentage)]		34 (54%)	6 (38%)	28 (60%)
Abdominal pain during 2-week run-in (mean NRS)		3.4 ± 1.8	3.0 ± 1.4	3.6 ± 1.9
Abdominal pain during 14-week treatment (mean NRS)		3.1 ± 1.7	1.7 ± 1.1*^/+^	3.5 ± 1.6
Fecal type during 2-week run-in (mean number)		4.6 ± 1.1	4.5 ± 1.8	4.7 ± 1.2
Fecal type during 14-week treatment (mean number)		4.5 ± 1.0	4.1 ± 1.6*	4.6 ± 1.1
Irritable bowel syndrome type				
Constipation predominant [n (percentage)]		0 (0%)	0 (0%)	0 (0%)
Diarrhea predominant [n (percentage)]		36 (57%)	11 (69%)	25 (53%)
Mixed [n (percentage)]		27 (43%)	5 (31%)	22 (47%)
Fecal organic acids	Visit	n = 51	n = 13	n = 38
Acetate (mmol/100 g of feces)	V2	3.4 ± 3.5	2.6 ± 1.7	3.6 ± 3.9
	V4	3.7 ± 3.1	3.0 ± 1.8	3.9 ± 3.4
Butyrate (mmol/100 g of feces)	V2	3.3 ± 2.9	3.5 ± 2.4	3.3 ± 3.1
	V4	3.7 ± 3.3	4.6 ± 3.5	3.5 ± 3.3
Propionate (mmol/100 g of feces)	V2	1.3 ± 1.1	1.4 ± 0.9	1.3 ± 1.1
	V4	1.5 ± 1.9	1.5 ± 1.0	1.5 ± 2.1
Valerate (mmol/100 g of feces)	V2	1.3 ± 1.2	1.4 ± 0.6	1.2 ± 1.3
	V4	1.4 ± 1.0	1.4 ± 1.0	1.3 ± 1.0
Isovalerate (mmol/100 g of feces)	V2	1.0 ± 0.8	0.9 ± 0.5	1.0 ± 0.9
	V4	1.0 ± 0.7	1.0 ± 0.8	1.0 ± 0.7
Lactate (mmol/1 kg of feces)	V2	2.2 ± 14.1	0.05 ± 0.10	2.9 ± 16.3
	V4	3.9 ± 24.4	0.2 ± 0.5	5.2 ± 28.2
Succinate (mmol/1 kg of feces)	V2	2.3 ± 7.7	0.3 ± 0.7	3.0 ± 8.9
	V4	5.5 ± 16.1	9.8 ± 26.0	4.0 ± 10.9
Serum markers	Visit	n = 61/51 ^1^	n = 16/14 ^1^	n = 47/37 ^1^
Citrulline (µg/ml)	V1	4.5 ± 1.2	4.5 ± 1.3	4.5 ± 1.1
	V4	4.5 ± 1.3	4.5 ± 1.2	4.5 ± 1.4
PV-1 (ng/ml)	V1	3.6 ± 1.6	3.7 ± 1.8	3.5 ± 1.6
	V4	4.7 ± 4.3	7.0 ± 6.7	3.8 ± 2.5
Zonulin (ng/ml)	V1	34.8 ± 8.6	32.6 ± 5.6	35.6 ± 9.4
	V4	36.0 ± 8.8	33.8 ± 5.6	36.8 ± 9.7
Alanine aminotransferase (U/l)	V1	20.1 ± 9.9	18.1 ± 7.1	20.7 ± 10.6
	V4	20.8 ± 10.3	19.6 ± 9.4	21.2 ± 10.7
Aspartate aminotransferase (U/l)	V1	19.8 ± 5.7	19.7 ± 6.5	19.8 ± 5.5
	V4	20.0 ± 6.5	18.7 ± 4.5	20.5 ± 7.0
Bilirubin (mg/dl)	V1	1.3 ± 3.1	1.0 ± 1.8	1.3 ± 3.4
	V4	1.1 ± 2.2	0.9 ± 1.3	1.2 ± 2.5
Alkaline phosphatase (U/l)	V1	62.2 ± 17.8	64.3 ± 19.9	61.5 ± 17.2
	V4	61.8 ± 16.5	64.0 ± 19.3	61.1 ± 15.6
Blood urea nitrogen (mg/dl)	V1	25.4 ± 10.4	26.2 ± 10.4	25.1 ± 10.5
	V4	25.5 ± 12.0	25.0 ± 10.8	25.7 ± 12.5
Creatinine (mg/dl)	V1	3.6 ± 15.8	5.7 ± 19.3	2.9 ± 14.6
	V4	4.1 ± 16.9	6.6 ± 21.4	3.2 ± 15.2

*Significantly different (*P* < 0.001) compared with run-in (baseline) in the R group.

**+**, significantly different (*P* < 0.0001) from abdominal pain after the 14-week treatment in the NR group.

^1^, the first and second numbers indicate “n” at V1 and V4, respectively.

### *The fecal abundance of* Collinsella aerofaciens *and other bacterial taxa may distinguish responder from non-responder NC-IBS patients*

**Figure 1.**
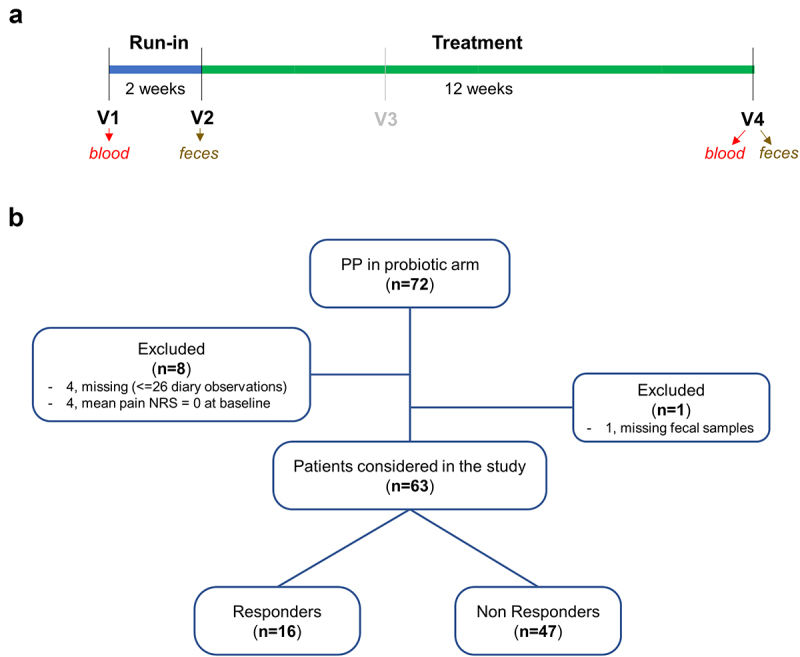
Study scheme (a) and summary of the IBS patients that concluded the PROBE-IBS/2 trial per protocol (PP) in the probiotic arm included in this study (b). NRS, numeric rating scale.

**Figure 2.**
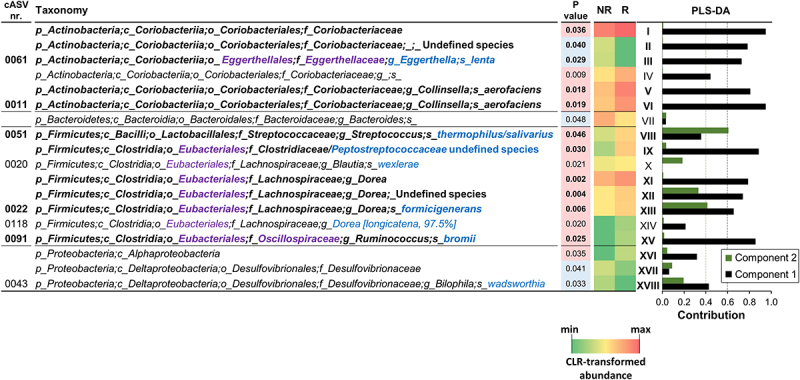
Fecal bacterial taxa distinguishing responder (R) from non-responder (NR) non-constipated IBS patients. *P* values, derived from Mann-Whitney tests on CLR-transformed bacterial abundances, are color-coded with red indicating taxa increased in the R group. The heatmap displays mean CLR-transformed abundances of reported taxonomic units. Taxonomic lineage is denoted: p, phylum; c, class; o, order; f, family; g, genus; s, species. Corrections to GreenGenes database nomenclature, based on NCBI Taxonomy, are shown in violet. Taxonomic names in blue were determined via manual BLASTN search in GenBank using corresponding read sequences. The histogram on the right illustrates the contribution of each bacterial taxon to the first two components of the PLSDA biplot in Figure 3, with Roman numerals linking taxa to the PLSDA loading plot. Bold text highlights the 11 bacterial taxa significantly contributing to variability in the PLSDA analysis.

**Figure 3.**
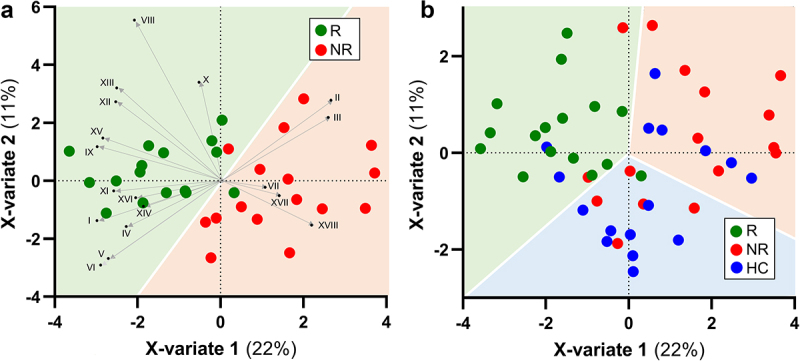
Biplot of PLS discriminant analysis (PLSDA) with prediction background for responder (R) and non-responder (NR) non-constipated IBS patients (panel a) and for R, NR, and healthy controls (HC) (panel b). Roman numerals in panel a refer to bacterial taxa in figure 2. The percentages indicate the explained variation at each axis. The Receiver Operating Characteristic (ROC) curves of the PLSDA model are shown in supplementary Figure S1.

**Figure 4.**
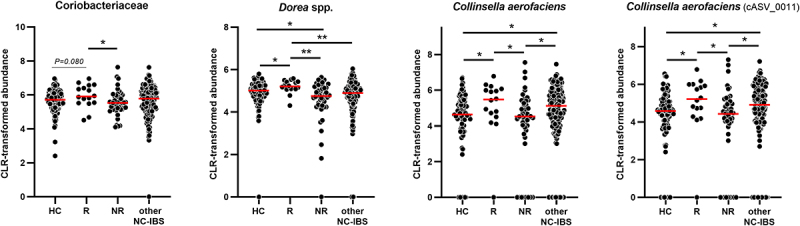
Dot plot of the most abundant bacterial taxa found to better discriminate between responders and non-responder patients in the PROBE-IBS/2 trial. HC, healthy controls (*n* = 100); R and NR, responder and non-responder NC-IBS patients in the PROBE-IBS/2 trial, respectively; other NC-IBS, other non-constipated IBS patients recruited at baseline during the PROBE-IBS/2 trial (*n* = 161); statistics is according to Mann-Whitney test; *, *P* < .05; **, *P* < .01.

R and NR NC-IBS patients were compared regarding fecal bacterial taxonomic structure at baseline (i.e., immediately before the beginning of the 12-week probiotic intake period; V2 in Figure 1a). Alpha- and beta-diversity analyses did not differentiate between the R and NR groups (not shown). In contrast, we found 18 bacterial taxa that were differentially represented between the two groups (Figure 2). Specifically, 13 taxa were overrepresented in the R group, including the following Gram-positive taxa belonging to the order Eubacteriales (formerly Clostridiales), genus *Dorea*, and species *Blautia wexlerae*, *Dorea formicigenerans*, *Dorea longicatena*, and *Ruminococcus bromii*. In addition, the abundance of the Actinobacteria species *Collinsella aerofaciens* and a bacterial unit of the *Streptococcus thermophilus*/*salivarius* taxonomic group increased in R patients at baseline, together with the Gram-negative class α-Proteobacteria and the Actinobacteria family Coriobacteriaceae. In contrast, only five bacterial taxa were overrepresented in the NR group, including *Eggerthella lenta* (class Coriobateriia), an undefined *Bacteroides* species, and *Bilophila wadsworthia* (class δ-Proteobacteria) (Figure 2). Subsequently, the fecal abundances of the bacteria found to be significantly different between R and NR patients were used in a PLS discriminant analysis (PLSDA). The resulting score plot significantly separated R patients from NR patients (Figure 3; Supplementary Figure S1). In addition, the PLSDA loading plot and contribution to component (CC) analysis revealed that 11 taxa contributed significantly to explaining the variability in the PLSDA model reported in Figure 3a: ten taxa for component 1 and two taxa for component 2, including *Dorea formicigenerans* (cASV0022), which contributed significantly to both components. In particular, the most relevant contributions to component 1 were provided by *Collinsella aerofaciens* cASV 0011 (CC = 0.95), the whole species *C. aerofaciens* (CC = 0.81), *Ruminococcus bromii* cASV 0091 (CC = 0.86), the family Coriobacteriaceae (CC = 0.95), the genus *Dorea* (CC = 0.79), and undefined Clostridiaceae/Peptostreptococcaceae species (represented mainly by reads ascribed to the Peptostreptococcaceae species *Terrisporobacter petrolearius*; CC = 0.89) (Figures 2 and 3a). These six taxa included the bacterial groups that discriminated R from NR, with the highest abundance in the fecal samples (see heatmap in Figure 2), viz., Coriobacteriaceae, *Dorea* spp., and *Collinsella aerofaciens*. Notably, the abundance of these bacterial taxa was higher in the R group than in the 100 healthy controls (Figure 4).

Subsequently, we performed PLSDA with the fecal abundances of the bacteria distinguishing R and NR patients, including the fecal microbiome data of the 100 healthy controls. Interestingly, this analysis revealed that R samples could be distinguished better from the other samples than NR samples from controls (0.91 prediction accuracy for R toward NR + controls; Figure 3b; Supplementary Figure S1).

### *The probiotic intervention reduced* C. aerofaciens *in responder patients*

**Figure 5.**
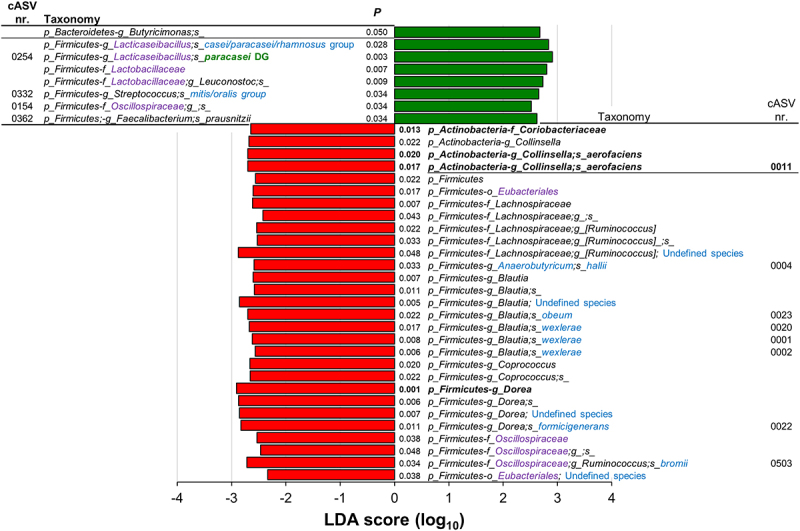
Linear discriminant analysis (LDA) effect size (LEfSe) graphics for responders (R) NC-IBS patients in the probiotic arm. LDA scores indicate taxa significantly (*P* < 0.05) higher before (V2; negative LDA) or after (V4; positive LDA) probiotic intake. Taxon levels are abbreviated: p, phylum; c, class; o, order; f, family; g, genus; and s, species. Corrections/updates to GreenGenes database nomenclature are marked in violet, while taxonomic names determined through a manual BLASTN search in GenBank using corresponding read sequences are highlighted in blue. Taxon cAVS 254 is identified as the administered probiotic strain (L. paracasei DG; highlighted in green).

Subsequently, we carried out LEfSe analysis on CLR-transformed taxonomic data using paired statistics to identify the bacterial taxa significantly modified by the probiotic intervention between the time points V2 and V4 in three groups of patients: R, NR, and R+NR. The taxon that increased more after probiotic intake in all three groups of samples was cASV 0254, which was ascribed to the *Lacticaseibacillus casei* group of species and plausibly corresponds to the probiotic strain administered in this study (*L. paracasei* DG). The results also revealed that probiotic intake induced a significant reduction in bacterial taxa, mainly in the R group (*n* = 29) compared to the NR (*n* = 13) and R+NR (*n* = 14) groups. In contrast, most of the taxa that significantly increased after probiotic intake were in the R+NR group (*n* = 25) compared to those in the NR (*n* = 16) and R (*n* = 8) groups (Figure 5 and Supplementary Figure S2). In R patients, a substantial reduction was observed in the majority of altered bacterial taxonomic units after probiotic administration. The affected taxa were predominantly affiliated with the order Eubacteriales, encompassing 24 out of 29 identified taxa. Notable species within this order included *Anaerobutyricum hallii*, *Blautia obeum*, *Blautia wexlerae*, *Dorea formicigenerans*, and *Ruminococcus bromii*. Notably, probiotic intervention induced a significant reduction in several taxonomic units reported above to contribute to the distinction between R and NR, *viz. Collinsella aerofaciens* (cASV 0011), *Blautia wexlerae* (cASV 0020), *Dorea formicigenerans* (cASV 0022), the genus *Dorea*, and the family Coriobacteriaceae (Figure 5). These taxa were not affected by probiotic intervention in NR patients (Supplementary Figure S2). Furthermore, qPCR experiments conducted on fecal DNA, utilizing species-specific primers, corroborated the heightened abundance of *C. aerofaciens* in R patients compared to NR counterparts. Importantly, the probiotic intervention reduced *C. aerofaciens* abundance in R patients (Supplementary Figure S3).

### *The analysis of a previous cohort of patients confirmed that* L. paracasei *DG intake may reduce* C. aerofaciens *and abdominal pain in non-constipated IBS*

**Figure 6.**
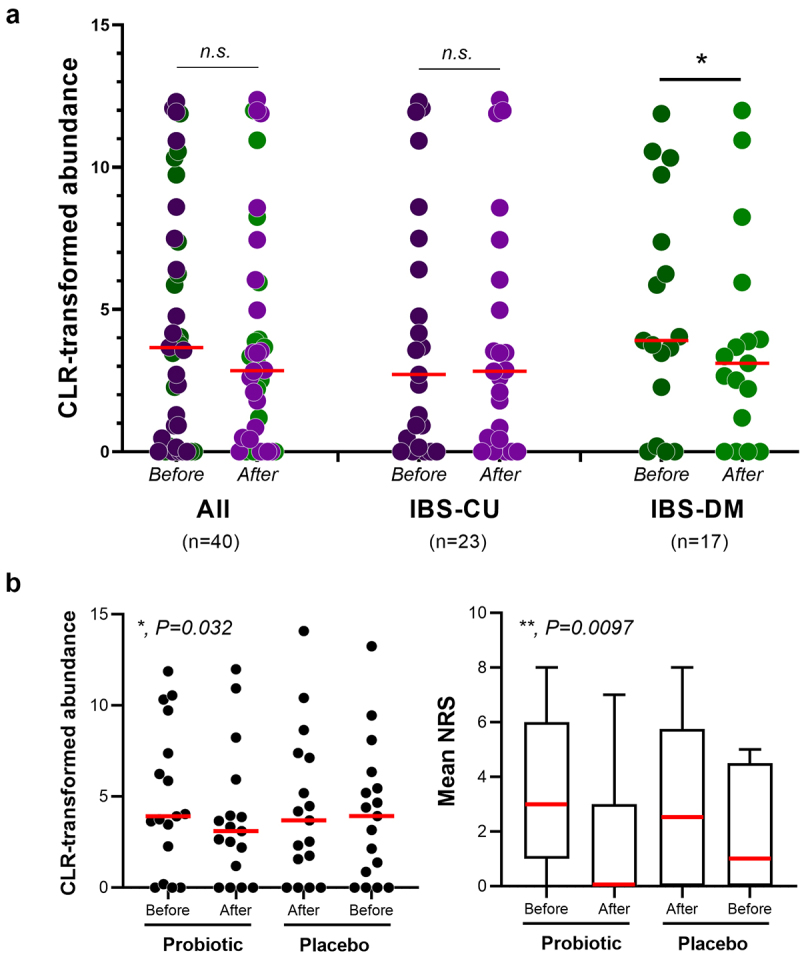
Analysis of data collected during the PROBE-IBS/1 cross-over trial. (a) fecal abundance of the bacterial species *collinsella aerofaciens* in IBS patients before and after the intake of the probiotic product. IBS-CU, constipated and undefined IBS patients; IBS-DM, diarrhea or mixed IBS patients. Statistics is according to Wilcoxon signed-rank test; *, *P* < .05; *n.S*., nonsignificant (*P* > .05). (b) fecal abundance of *C. aerofaciens* (on the left) and abdominal pain/discomfort (on the right) in IBS-DM patients during the PROBE-IBS/1 trial. NRS, numeric rating scale used to measure abdominal pain/discomfort. Statistics is according to non-parametric repeated measure ANOVA-Type statistic (RM-ATS). The reported *P* values indicate significant Time×Treatment interaction. **, *P* < .01; *, *P* < .05.

The results described above suggest that the fecal abundance of *C. aerofaciens* can predict the clinical efficacy of probiotic treatment with *L. paracasei* DG. To preliminarily confirm this hypothesis, we reconsidered the data of a previous clinical study, which was carried out using the same probiotic formulation (Enterolactis^Ⓡ^ PLUS) in a population of IBS patients (*n* = 40; PROBE-IBS/1; multicenter, randomized, double-blind, cross-over, 18-week, placebo-controlled, pilot trial;^20^ Analysis of the entire population of data (comprehensive IBS-C, IBS-U, IBS-M, and IBS-D) did not reveal any significant changes in *C. aerofaciens* abundance upon probiotic administration. Nonetheless, when the analysis was performed exclusively in IBS-D and IBS-M patients (*n* = 17), we observed a significant decrease in the abundance of *C. aerofaciens* (Figure 6a). Notably, compared to placebo intake, *L. paracasei* DG administration not only decreased *C. aerofaciens* abundance but also induced a significant reduction in abdominal pain [*P* < .01, according to non-parametric repeated measure ANOVA-Type Statistic (RM-ATS) (Figure 6b)]. In contrast, abdominal pain did not change significantly in patients with IBS-C and IBM-U (*P* = 0.34 according to RM-ATS). Finally, when we removed the participants from the analysis to the PROBE-IBS/1 that had an increase in the levels of *C. aerofaciens*, abdominal pain significantly decreased in the population of patients independent of IBS type (*n* = 16; *P* = .027), whereas abdominal pain did not change significantly in the other patients (*n* = 14; *P* = .540) (not shown), suggesting that the variation in *C. aerofaciens* can be inversely associated with the change in abdominal pain in IBS.

### The fecal abundance of *C. aerofaciens* is associated to PV-1 serum levels

**Figure 7.**
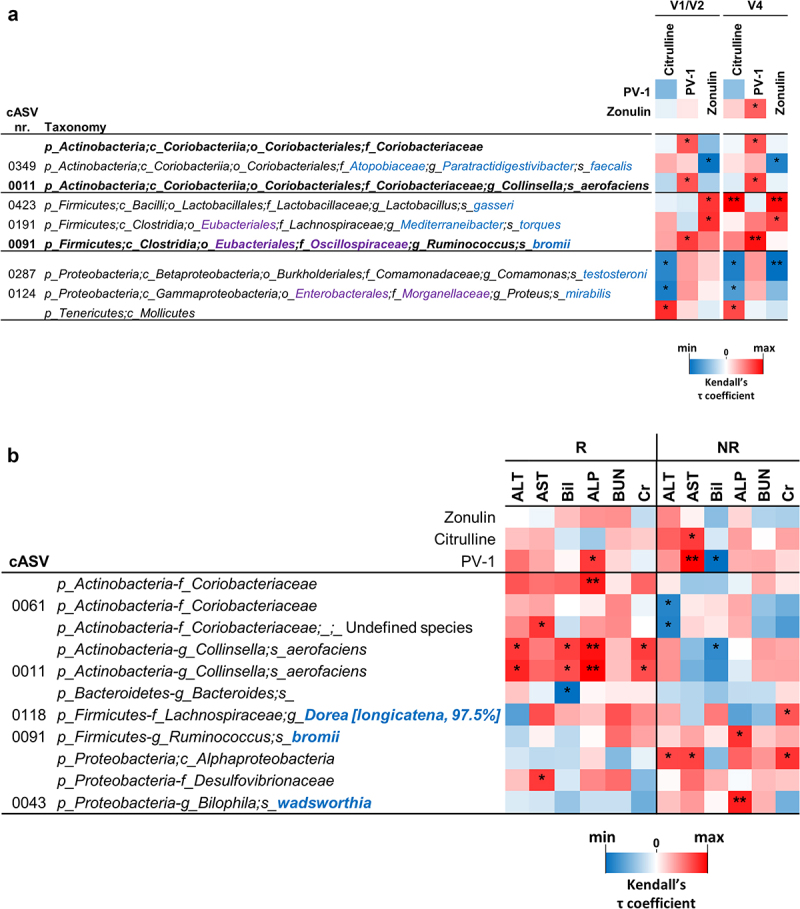
Correlation analyses between bacterial taxa and serum markers for permeability (panel a) and liver and kidney functionality (panel b). In panel a, correlation analyses were conducted with data from blood samples collected before run-in (visit V1) and fecal samples collected before the probiotic intervention (visit V2), as well as with data from blood and fecal samples collected at the end of the probiotic intervention (visit V4) denoted as V1/V2 and V4, respectively. Only taxa exhibiting consistent significant correlations at both V1/V2 and V4 are displayed. The heatmap represents the τ coefficient of Kendall rank correlation (*, *P* < .05; **, *P* < .01; ***, *P* < .001). Taxonomic lineage is indicated as follows: p, phylum; c, class; o, order; f, family; g, genus; s, species. Corrections/updates to GreenGenes database nomenclature are highlighted in violet, and taxonomic names determined through a manual BLASTN search are in blue. ALT, alanine aminotransferase; AST, aspartate-aminotransferase; Bil, bilirubin; ALP, alkaline phosphatase; BUN, blood urea nitrogen; cr, creatinine.

In subsequent experiments, we performed a correlation analysis between the fecal abundance of bacterial taxa and serum markers of intestinal function and permeability, viz. citrulline, PV-1, and zonulin. Correlation analysis was first performed with all available data collected from PROBE-IBS/2 participants at baseline (*n* = 191). For baseline measurements, serum samples were collected at V1, that is, 2 weeks before the collection of fecal samples (V2; see the study scheme in Figure 1). Blood and fecal samples were collected on the same day at the end of the probiotic intervention (visit V4). To avoid possible errors due to collection time discrepancy (V1 for blood and V2 for feces), correlation analysis was performed with data from samples collected at V4 (*n* = 177). The results revealed a significant correlation between serum parameters and numerous bacterial taxa (*n* = 88 at V1/V2 and *n* = 123 at V4; Supplementary Figure S4). Nonetheless, only nine taxa displayed the same significant associations at V1/V2 and V4 time points. Specifically, we found significant correlations with serum zonulin levels for *Lactobacillus gasseri* (cASV 0423), *Mediterraneibacter torques* (cASV 0191) (positive correlation), and *Paratractidigestivibacter faecalis* (cASV 349) (negative correlation). In addition, three taxa were found to correlate with citrulline: *Comamonas testosteroni* (cASV 287), *Proteus mirabilis* (cASV 287) (negative correlation), and the class Mollicutes (positive correlation) (Figure 7). Notably, the other three taxa were the same as those reported above among the four bacterial groups that better discriminated between R and NR patients (i.e., *C. aerofaciens* cASV 0011, *R. bromii* cASV 0091, and the family Coriobacteriaceae), all of which showed a significant positive correlation with the serum marker PV-1 (Figure 7a).

Finally, we used in the correlation analysis the data referred to serum markers of liver and kidney functioning, which were available for R and NR patients at baseline. The results showed that only for R-associated data, both serum PV-1 and *C. aerofaciens* were significantly correlated with the serum levels of liver alkaline phosphatase (Figure 7b). In addition, *C. aerofaciens* was positively correlated with other serum markers, *viz*. alanine aminotransferase, bilirubin, and creatinine (Figure 7b).

## Discussion

IBS is a multifactorial intestinal functional disorder characterized by abdominal pain and altered bowel habits.^21^ IBS is largely heterogeneous in etiology, pathophysiology, symptom manifestation, and severity, and also within the same subtype.^21^ As a plausible consequence, attempts to identify effective objective biomarkers for IBS are disappointing.^22^ The absence of effective biomarkers suggests that IBS patients, in addition to conventional subtyping based on bowel habits, could be further stratified according to other host- or microbiome-associated biomarkers. In addition, person-specific responses to probiotic effects have been reported, supporting the need for personalized probiotic approaches.^23^ In this context, we searched for potential biomarkers characterizing IBS patients who had significant clinical improvement after taking the *L. paracasei* DG probiotic within the PROBE-IBS/2 trial.

Several serum markers failed to distinguish clinical responders (R) from non-responders (NR), whereas significant discrimination was obtained according to the abundance of several bacterial taxa. The potential value of the microbiome structure in predicting the response to therapeutic treatment in IBS was recently confirmed by Vervier et al., who found that IBS patients who had significant clinical benefits from a low FODMAP diet regimen were characterized by a distinct microbiome structure, depleted in Bacteroidetes, and enriched in Firmicutes and genes for amino acid and carbohydrate metabolism.^24^ In particular, the authors defined the distinct microbiota profiles as “pathogenic-like,” in opposition to the microbiota of patients who did not respond to the low FODMAP diet, which was defined as “health-like”.^24^

Notably, the bacterial taxonomic differences that distinguish R from NR patients also distinguished R patients from healthy controls. In contrast, the bacterial taxonomic differences between NR patients and healthy controls were relatively limited. We can, therefore, hypothesize that the R patients in the PROBE-IBS/2 trial possess an altered (potentially dysbiotic) fecal microbiota. This speculation is supported by the information available in the scientific literature concerning the role of the most abundant discriminative bacterial taxa found in our study: the family Coriobacteriaceae, genus *Dorea*, and species *Collinsella aerofaciens*. Several studies have reported that the family Coriobacteriaceae is overrepresented in the intestinal microbiota of IBS patients.^25^ In addition, *Dorea* spp. and *C. aerofaciens* were found to be enhanced in IBS compared to healthy controls, specifically in diarrhea-predominant IBS.^26–28^ Notably, *Dorea* spp. and *C. aerofaciens* produce hydrogen (H_2_), ethanol, and formate as the main end-products of their glucose metabolism.^29,30^ Hydrogen is one of the most abundant gases produced by bacteria in the human colon,^31^ and has been shown to reduce colonic transit.^32^ Accordingly, intestinal hydrogen production is associated with IBS,^33^ particularly in NC-IBS patients.^34,35^

Coriobacteriaceae, *Blautia* spp., and *C. aerofaciens* have been associated with various noncommunicable diseases and dysfunctional metabolic conditions. For instance, the abundance of the family Coriobacteriaceae was reported to be increased in patients with type 2 diabetes^36^ and IBD (both Crohn’s Disease and Ulcerative Colitis)^37^ compared to healthy controls. Furthermore, the Eubacteriales genus *Dorea*, which was reported to be increased in multiple sclerosis patients, was demonstrated to include species that promote inflammation by triggering IFN-γ production and to potentially enhance intestinal permeability by degrading mucin and metabolizing sialic acids.^38,39^ In addition, Coriobacteriaceae, *Blautia* spp., and *C. aerofaciens* were all enhanced in overweight/obese subjects.^40^ In addition, the authors of this study reported a positive association of *Dorea* spp. and *C. aerofaciens* with anthropometric parameters (waist circumference) and proposed these bacteria as obesity biomarkers.^40^

The available literature indicates *C. aerofaciens* as a pathobiont, which is also overabundant in metabolic syndrome,^41^ type 2 diabetes,^42^ autoimmune polyendocrine syndrome type 1,^43^ and psoriatic arthritis (together with the species *Dorea formicigenerans*).^44^ Furthermore, *C. aerofaciens* exacerbates arthritis in a collagen-induced arthritis model by increasing gut permeability and triggering the expression of IL-17, CXCL1, and CXCL5 in intestinal epithelial cells.^45^

Interestingly, in our study, probiotic intervention with *L. paracasei* DG significantly reduced the fecal abundance of Coriobacteriaceae, *Dorea* spp., and *C. aerofaciens*. Moreover, a significant decrease in the genus Coriobacteriaceae and species *C. aerofaciens* after treatment with the probiotic *L. paracasei* DG was also observed by re-analyzing the data collected during a previous intervention trial involving a different group of NC-IBS patients. Furthermore, in our study, *C. aerofaciens* and its entire family *Coriobacteriaceae* were found to positively correlate with plasma vesicle-associated protein-1 (PV-1) and alkaline phosphatase (ALP) serum levels. Simultaneously, PV-1 and ALP positively correlated with each other. PV-1 is a transmembrane glycoprotein expressed on blood vessels and lymphatic endothelial cells, which regulates endothelial permeability.^46,47^ The increased expression of PV-1 in endothelial cells was found to correlate with the systemic dissemination of enteropathogens and, notably, with higher serum levels of alanine transaminase (ALT),^48^ indicators of liver damage, similar to ALP. Accordingly, we found that *C. aerofaciens*, in addition to ALP, was also correlated with the serum levels of the liver function markers ALT and bilirubin. These results suggest that the bacterial community structure of the fecal microbiota in R NC-IBS patients, which is enriched in potentially pro-inflammatory bacteria, may detrimentally influence the gut-liver axis by promoting endothelial leakiness, consequently resulting in the alteration of liver functionality. In support of this hypothesis, recent studies have suggested a potential association between IBS and elevated ALT and metabolic syndrome,^49^ and the association of a high degree of nonalcoholic fatty liver with an increased risk of IBS.^50^ In support of this hypothesis, Chen et al. demonstrated that *C. aerofaciens* may significantly decrease the expression of zonula occludens-1 (ZO-1),^45^ a tight junction protein also demonstrated to control endothelial adherens junctions.^51^

This study has several limitations, the most important of which is the limited number of responder NC-IBS patients that we could include in the analyses. In addition, the intestinal microbial ecosystem was exclusively studied through 16S rRNA gene profiling, which does not provide information on strain- or biotype-specific microbial functions. Owing to functional redundancy, the ecosystem services of human-associated microbiomes are more stable than their corresponding taxonomic community structures, which also express geographical/racial variations,^52^ potentially limiting the generalizability of the results of our study. On the other hand, one strength of this study is that the obtained results concerning *C. aerofaciens* and the efficacy of the probiotic formulation have also been confirmed with a different dataset generated in a previous independent trial. Furthermore, we found solid scientific literature supporting this hypothesis. Finally, another advantage of this study is that it involved the recruitment of subjects from 20 different centers, allowing the identification of a sufficiently large number of subjects to identify the subgroup of patients with dysbiotic IBS and to increase the generalizability of the obtained results.

Another limitation of this study is the need for more dietary information for the patients during the study. Therefore, it cannot be ruled out that the consumption of certain foods may have influenced the response to probiotic treatment.

## Conclusion

In this study, we proposed that a 12-week intake of the probiotic bacterium *Lacticaseibacillus paracasei* DG can be clinically effective in a subgroup of non-constipated IBS patients characterized by an altered fecal microbiota (dysbiotic NC-IBS patients), which resembles that observed in metabolic syndrome-associated pathologic or pre-pathologic conditions. Among the putative pathobionts increased in patients with dysbiotic NC-IBS, *Collinsella aerofaciens*, a bacterium reported to contribute to pro-inflammatory immune states, was positively associated with markers of increased endothelial permeability and liver functionality, suggesting the involvement of the gut-liver axis in this subgroup of IBS patients. An analysis of the fecal microbiota focused on these bacteria could permit the identification of NC-IBS patients who can obtain a significant clinical benefit from the probiotic treatment assessed during the PROBE-IBS/2 trial.

## Patients and methods

### Study design and population

The volunteers and samples in this study were derived from a multicenter, randomized, double-blind, parallel-group, placebo-controlled clinical trial (PSC-DS PROBE2-IBS/17, PROBE-IBS/2), which was carried out as described in ClinicalTrials.gov under the identifier NCT03449628. Eligible patients with symptoms meeting the Rome IV criteria for the diagnosis of IBS without constipation (i.e., patients with IBS-D and IBS-M) were recruited from 20 Italian centers. The inclusion and exclusion criteria are reported in Supplementary Methods (Data supplement file).

This study was primarily based on the analysis of patients that concluded the PROBE-IBS/2 trial per protocol in the probiotic arm (*n* = 72) (Figure 1). Nine of them were excluded due to missing samples (*n* = 1), missing data for the calculation of the composite endpoint (*n* = 4), and no pain reported during the 2-week run-in period (i.e., mean numeric rating scale [NRS] = 0; *n* = 4) (Figure 1). Consequently, 63 patients were included in this study, with a mean age of 35 ± 12 years and sex distribution of 34:29 F/M (Table 1). In addition, we also considered samples collected during PROB-IBS/2 at V2 from 172 patients with NC-IBS and 100 healthy controls. The baseline characteristics of the patients are summarized in Table 1.

### Ethics approval

The protocol was approved by the Ethics Committee of the coordinating center (Hospital S. Orsola Malpighi-Bologna; approval identification no:67/2017/U/Sper on June 13, 2017) and by the Ethics Committee of each participating site. The trial was conducted in compliance with the Declaration of Helsinki and principles of good clinical practice. Written informed consent was obtained from all the participants.

### Probiotic product used in the trial

L. casei DG^Ⓡ^ (*Lacticaseibacillus paracasei* DG I1572, DSM 34,154) was administered with the formulation Enterolactis^Ⓡ^ PLUS, consisting of at least 24 billion CFU in a 405 mg hydroxypropyl cellulose capsule (two capsules per day ingested with still water in an empty stomach). Viable counts on a representative number of capsules were performed before and after the trial by spreading serial dilutions on de Man-Rogosa-Sharpe (MRS) agar (Difco Laboratories Inc., Detroit, MI). Each capsule contained an average of 4.1 × 10^10^ bacterial cells per capsule at the beginning of the study, according to the total bacterial cell count performed by cytofluorimetry (BD Accuri C6; Becton Dickinson Italia, Milan, Italy) upon SYBR green cell labeling. The capsules also contain silicon dioxide and fatty acid magnesium salts as anti-caking agents.

### Analysis of the fecal microbial ecosystem

The bacterial community structure of fecal samples was studied through metataxonomics by 16S rRNA gene profiling up to the level of amplicon sequence variants (ASV) clustered at 97% similarity (cASV) as reported in the supplementary methods (Data supplement file). The following organic acids were detected in fecal samples: acetate, butyrate, propionate, valerate, isovalerate, lactate, and succinate. These molecules were detected and quantified in fecal samples by Ultra-Performance Liquid Chromatography – High-Resolution Mass Spectrometry (UPLC-HR-MS) on an Acquity UPLC separation module (Waters, Milford, MA) coupled with an Exactive Orbitrap MS using a HESI-II probe for electrospray ionization (Thermo Scientific, San Jose, CA), as previously described in detail^28^.

### Collinsella aerofaciens *quantification through quantitative PCR*

The *Collinsella aerofaciens* species was quantified in fecal samples using a TaqMan real-time quantitative PCR protocol developed in this study as described in detail in the supplementary methods (Data supplement file).

### Analysis of serum samples

Serum samples were used for the quantification of citrulline and markers of permeability (zonulin and PV-1), liver [alanine aminotransferase (ALT), aspartate aminotransferase (AST), bilirubin (Bil), alkaline phosphatase (ALP)], and kidney [blood urea nitrogen (BUN) and creatinine (Cr)] functionality, as described in the Supplementary Methods (Data supplement file).

### Data analysis, bioinformatics, and statistics

Collected data were analyzed after separating the patients into two groups: responders (R) and non-responders (NR). R and NR were defined according to the primary endpoint of PROBE-IBS/2, which was based on the composite response over 12 weeks. In detail, a patient was considered R when recorded on ≥ 50% of the days, a reduction of ≥ 30% from their average baseline score for their worst abdominal pain, and on the same days, a stool consistency score ≤ 5.^53^ The standard 11-point numeric rating scale (0 = none to 10 = worst possible pain) was used to measure abdominal pain, and the stool form was measured using the Bristol Stool Form Scale (BSFS).

Statistical calculations, including partial least squares discriminant analysis (PLSDA), were performed using R programming language (version 3.4.2). Read abundances underwent *centered log-ratio transformation* (CLR). Differently abundant taxa between the groups were identified using linear discriminant analysis (LDA) combined with the effect size (LEfSe) algorithm^54^ on CLR-transformed taxonomic abundances. A cutoff value of LDA score (log10) above 2.0 was chosen. For paired/unpaired matches, paired/unpaired Student’s t test or Mann-Whitney U and Wilcoxon Signed-Ranks tests were adopted depending on normal distribution assessed through Shapiro-Francia test. A non-parametric repeated measures ANOVA-Type Statistic (RM-ATS) was also used where appropriate. Correlation analyses were performed by calculating Kendall’s τ rank correlation coefficient.

## Supplementary Material

Data supplement file_Guglielmetti_R1_CLEAN.docx

## Data Availability

Metataxonomic raw sequencing data are available as FASTQ files in the European Nucleotide Archive (ENA) of the European Bioinformatics Institute under the accession code PRJEB56302. Metataxonomic raw sequencing data related to PROBE-IBS/1^20^ are under accession code PRJEB18753. Processed data are included in the article or uploaded as supplementary materials. All the other data are available upon request.

## Collaborators

Bruno Annibale, Roma; Guido Basilisco, Milano; Leonilde Bonfrate, Bari; Michele Cicala, Roma; Rocco Cosintino, Roma; Antonio Di Sabatino, Pavia; Raffaella Ferraro, Vercelli; Francesca Galeazzi, Padova; Bastianello Germanà, Belluno; Giovanni Maconi, Milano; Santino Marchi, Pisa; Gerardo Nardone, Napoli; Matteo Neri, Chieti; Fabio Pace, Seriate; Piero Portincasa, Bari; Franco Radaelli, Como; Marcello Rodi, Vercelli; Giovanni Sarnelli, Napoli; Paolo Usai, Cagliari.
