# Supplementary material for: Collinsella aerofaciens as a predictive marker of response to probiotic treatment in non-constipated irritable bowel syndrome
Source: Gut Microbes. 2024 Jan 4;16(1):2298246. doi: 10.1080/19490976.2023.2298246 (PMC10773624; doi:10.1080/19490976.2023.2298246)

# Supplementary methods

*Inclusion criteria*

Patients have been included in the study if they meet all the following criteria:

- Age > 18 years and ≤ 65 years.
- A positive diagnosis of nonconstipated IBS (i.e., IBS-D, and IBS-M, both males and females), according to Rome IV criteria.
- A negative outcome of colonoscopy performed within 5 years before screening if patient is at least 50 years old, or if patient meet any of the following alarm features:

1. Has a documented weight loss within the past 6 months; or
2. Has nocturnal symptoms; or
3. Has a familiar history of colon cancer; or
4. Has blood mixed with their stool (excluding blood from hemorrhoids)

- Negative relevant additional screening or consultation whenever appropriate.
- Ability of conforming to the study protocol.

*Exclusion criteria*

Patients have been excluded from this study if they meet any of the following criteria:

- Patients with IBS-C or IBS-U according to Rome IV criteria (see Table 1).
- Presence of any relevant organic, systemic or metabolic disease (particularly significant history of cardiac, renal, neurological, psychiatric, oncology, endocrinology, metabolic or hepatic disease), or abnormal laboratory values detected during the run-in period that will be deemed clinically significant on the basis of predefined values, i.e., liver or kidney functional levels 2-times greater that the upper reference levels.
- Ascertained intestinal organic diseases, including ascertained celiac disease or inflammatory bowel diseases (Crohn's disease, ulcerative colitis, diverticular disease, infectious colitis, ischemic colitis, microscopic colitis).
- Previous major abdominal surgeries.
- Active malignancy of any type, or history of a malignancy (patients with a history of other malignancies that have been surgically removed and who have no evidence of recurrence for at least five years before study enrolment are also acceptable).
- Untreated food intolerance such as ascertained or suspected lactose intolerance, as defined by anamnestic evaluation or, if appropriate, lactose breath test.
- Assumption of probiotics or topic and/or systemic antibiotic therapy during the last month.
- Systematical/frequent assumption of contact laxatives.
- Presence of red or white flags at the Rome IV Psychosocial Alarm Questionnaire for Functional Gastrointestinal Disorders (see Appendix 4)
- Females of childbearing potential, in the absence of effective contraceptive methods.
- Pregnant women.
- Inability to conform with protocol.
- Treatment with any investigational drug within the previous 30 days.
- Recent history or suspicion of alcohol abuse or drug addiction.

*Characteristics of the participants*

The characteristics of the IBS patients included in the study. Sex: 1, male; 2, female. Ethnicity: 1, Caucasian. Responder: 0, no; 1, yes.

| **Patient code** | **Age (years)** | **Sex** | **Ethnicity** | **IBS type** | **Responder** |
| --- | --- | --- | --- | --- | --- |
| IP001 | 29 | 2 | 1 | D | 0 |
| IP002 | 38 | 1 | 1 | M | 1 |
| IP003 | 54 | 1 | 1 | D | 0 |
| IP004 | 46 | 1 | 1 | M | 0 |
| IP005 | 23 | 1 | 1 | D | 0 |
| IP006 | 47 | 1 | 1 | D | 0 |
| IP007 | 29 | 1 | 1 | M | 0 |
| IP008 | 54 | 2 | 1 | D | 0 |
| IP009 | 46 | 2 | 1 | D | 0 |
| IP010 | 29 | 1 | 1 | D | 0 |
| IP011 | 28 | 1 | 1 | M | 0 |
| IP012 | 29 | 2 | 1 | D | 0 |
| IP013 | 28 | 2 | 1 | D | 0 |
| IP014 | 21 | 2 | 1 | M | 0 |
| IP015 | 46 | 1 | 1 | D | 0 |
| IP016 | 51 | 2 | 1 | M | 1 |
| IP017 | 28 | 2 | 1 | D | 0 |
| IP018 | 33 | 1 | 1 | D | 0 |
| IP019 | 59 | 1 | 1 | M | 0 |
| IP020 | 59 | 2 | 1 | D | 0 |
| IP021 | 26 | 1 | 1 | D | 0 |
| IP022 | 26 | 1 | 1 | D | 1 |
| IP023 | 40 | 1 | 1 | M | 0 |
| IP024 | 25 | 2 | 1 | M | 0 |
| IP025 | 25 | 1 | 1 | D | 1 |
| IP026 | 26 | 2 | 1 | M | 0 |
| IP027 | 24 | 1 | 1 | M | 0 |
| IP028 | 24 | 1 | 1 | D | 1 |
| IP029 | 26 | 2 | 1 | D | 0 |
| IP030 | 25 | 1 | 1 | D | 0 |
| IP031 | 30 | 1 | 1 | M | 1 |
| IP032 | 29 | 1 | 1 | D | 1 |
| IP033 | 24 | 1 | 1 | M | 0 |
| IP034 | 31 | 1 | 1 | M | 0 |
| IP035 | 25 | 2 | 1 | M | 0 |
| IP036 | 24 | 2 | 1 | M | 0 |
| IP037 | 31 | 1 | 1 | M | 1 |
| IP038 | 31 | 1 | 1 | M | 0 |
| IP039 | 46 | 1 | 1 | D | 1 |
| IP040 | 24 | 2 | 1 | M | 1 |
| IP041 | 44 | 1 | 1 | D | 0 |
| IP042 | 65 | 2 | 1 | M | 0 |
| IP043 | 38 | 2 | 1 | M | 0 |
| IP044 | 52 | 2 | 1 | D | 0 |
| IP045 | 21 | 2 | 1 | D | 1 |
| IP046 | 23 | 2 | 1 | D | 0 |
| IP047 | 28 | 2 | 1 | M | 0 |
| IP048 | 35 | 2 | 1 | D | 1 |
| IP049 | 21 | 2 | 1 | M | 0 |
| IP050 | 27 | 2 | 1 | M | 0 |
| IP051 | 55 | 2 | 1 | D | 0 |
| IP052 | 35 | 2 | 1 | M | 0 |
| IP053 | 48 | 1 | 1 | D | 1 |
| IP054 | 27 | 2 | 1 | D | 0 |
| IP055 | 52 | 2 | 1 | D | 1 |
| IP056 | 60 | 2 | 1 | D | 0 |
| IP057 | 22 | 2 | 1 | M | 0 |
| IP058 | 58 | 2 | 1 | D | 0 |
| IP059 | 31 | 1 | 1 | D | 1 |
| IP060 | 30 | 1 | 1 | D | 0 |
| IP061 | 20 | 2 | 1 | D | 1 |
| IP062 | 19 | 2 | 1 | D | 0 |
| IP063 | 42 | 2 | 1 | M | 0 |

*Taxonomic proﬁling of fecal bacterial communities*

Total DNA was extracted from 150 mg of feces using a QIAsymphony PowerFecal Pro DNA Kit (Qiagen, Milan, Italy) following the manufacturer's instructions. The extracted DNA was checked for quality by measuring absorbance at 260/280 nm and 260/230 nm and was quantified through the Qubit Broad Range kit (Thermo Fisher Scientific, Waltham, MA, USA). NovaSeq 6000, 2x250bp (NovaSeq 6000 SP Reagent Kit, 500 cycles) was used to sequence the 16S rRNA gene amplicons encompassing the V3 and V4 variable regions obtained with primers 341F (5’-CCT ACG GGN GGC WGC AG-3’) and 805R (5’-GAC TAC HVG GGT ATC TAA TCC-3’) (LC Sciences, Houston, TX). The obtained sequencing reads were managed with the bioinformatic pipeline Quantitative Insights Into Microbial Ecology (QIIME) 2 version 2022.2 through the Divisive Amplicon Denoising Algorithm (DADA2; ^1^) adopting the Greengenes database v. 13_8 for taxonomic assignment to amplicon sequence variants (ASVs) clustered at a 97% similarity (cASV). To limit technological bias, the 16S rRNA gene profiling analysis has been carried out simultaneously with all fecal samples considered in this study (including the 100 samples from healthy volunteers). Metataxonomic data used in this study are available as FASTQ data in the European Nucleotide Archive (ENA) of the European Bioinformatics Institute under accession code PRJEB56302.

*Quantification of the species* Collinsella aerofaciens *through quantitative PCR*

The *Collinsella aerofaciens* species was quantified using TaqMan real-time quantitative PCR on DNA extracted from fecal samples. The target region of the *C. aerofaciens* genome for designing species-specific primers and probe was identified through a pangenome analysis using Prokka ^2^ and Roary ^3^. Specifically, the region ATP54890.1, encoding a hydrogenase, was chosen, as it was found to be unique to the genomes of *C. aerofaciens* species and not present in genomes of other *Collinsella* species. For the analysis, *Collinsella* genomes were retrieved from the NCBI Genome database (<https://www.ncbi.nlm.nih.gov/datasets/genome/?taxon=102106>). The designed primers and probe were as follows:

Forward Primer: 5’-TAACGGACACCACGAACAGATGCTC-3’

Reverse Primer: 5’-ACAGCCTATCCACGTCCAAGTC-3’

TaqMan probe with a dye label (FAM): 5’-CAGATGTTCGGCGCTATTGCCAAGACGT-3’

Amplicon length: 111 bp

The specificity of the primers and probe was also verified through BLASTN searches in the GenBank database. The region used to design the BactQuant (panbacterial) assay falls within the V3 and V4 regions of the prokaryotic 16S rRNA gene and was adopted from Liu et al.^4^. Reactions were carried out using the Sso Advanced Universal probe supermix (Bio-Rad). The thermal protocol included an initial denaturation phase at 95°C for 2 minutes, followed by 40 cycles of denaturation at 95°C for 15 seconds and annealing/extension at 60°C for 60 seconds. Quantification was expressed as the relative amount of the target (*C. aerofaciens*) compared to the panbacterial quantification using the DeltaCq method with the Bio-Rad CFX Maestro program.

*Organic acids quantification*

Organic acids (acetate, butyrate, propionate, valerate, isovalerate, lactate, and succinate) were detected and quantified in fecal samples by Ultra-Performance Liquid Chromatography–High-Resolution Mass Spectrometry (UPLC-HR-MS) on Acquity UPLC separation module (Waters, Milford, MA) coupled with an Exactive Orbitrap MS through a HESI-II probe for electrospray ionization (Thermo Scientiﬁc, San Jose, CA), as previously described in detail ^5^.

*Citrulline quantification*

Serum samples were used for the quantification of the intestinal functionality marker citrulline by high resolution mass spectrometry analysis. Serum samples were thawed on ice and diluted in 1:4 with cold acetonitrile and added with L-Citrulline-d4 as internal standard (IS) at a concentration of 25 μg/ml. Samples have been then centrifuged at 14800 rpm × 20 min at 4°C and supernatant was then collected in glass vial and stored at 4°C until analysis. All samples have been analysed at UNITECH OMICs (University of Milano, Italy) using ExionLCTM AD system (SCIEX) connected to TripleTOFTM 6600 System (SCIEX) equipped with Turbo VTM Ion Source with ESI Probe. Chromatographic separation was achieved on Kinetex® HILIC (Phenomenex®), 100 mm (Length) x 2.1 mm (ID) x 1.7 μm (Particle Size), equipped with a pre-column, using mobile phase A (2 mM Ammonium Formate in water, 0.2% formic acid) and mobile phase B (2 mM Ammonium Formate in acetonitrile, 0.2% formic acid) at a flow rate of 400 μl/min. For each batch of analysis, a standard sample at a concentration of 25 μg/ml containing L-Citrullina-d4 (IS) was injected. A calibration curve was constructed by using L-citrulline freshly prepared for each batch of analysis. The column and autosampler temperatures were set at 45°C and 7°C respectively. The sample injection volume was 5 μl. MS spectra were collected over an m/z range of 50-500 Da, operating in PIS (Product Ion Scan) of 180.1 for L-Citrulline-d4 [M+H]+ and 176.1 for L-Citrulline [M+H]+. Collision energy was set at 35 (CES 25). Polarity: positive.

*Zonulin and PV-1 quantification*

The intestinal permeability marker zonulin was quantified in serum samples as previously described ^6^ using an IDK^®^ Zonulin ELISA Kit (Immundiagnostik AG, Germany). This assay is based on a competitive Elisa method with addition to standard and control samples of a biotinylate zonulin tracer and the subsequent use of a pre-coated multiwell plate with polyclonal anti-zonulin antibody. Peroxidase-labelled streptavidin addition was used to bind the biotinylate zonulin tracer and after the reaction, absorbance at 450 nm was measured by means of an Eon plate reader (BioTek-Agilent, Santa Clara, CA). Serum zonulin concentrations were quantified using a standard curve calculated by a 4-parameter algorithm as reported by the manufacturer. The endothelial permeability marker plasmalemma vesicle associated protein (PLVAP)/PV-1 was measured in serum samples by means of the Human PVLAP ELISA Kit (Fine test, China). Samples were processed according to manufacturers’ instruction and absorbance at 450 nm was measured using an Eon plate reader. Absorbance data were then interpolated by means of a logarithmic standard curve, which was calculated for each single plate analyzed.

*Analysis of liver and kidney functionality markers*

The following markers of liver and kidney functionality were assessed in serum samples: alanine aminotransferase (ALT), aspartate-aminotransferase (AST), bilirubin (Bil), alkaline phosphatase (ALP), blood urea nitrogen (BUN), and creatinine (Cr). AST and ALT were assessed by enzymatic dosage without activation by pyridoxal phosphate (Cobas 8000 - Roche Diagnostics). Bil and ALP were dosed by enzymatic colorimetric test (Cobas 8000 - Roche Diagnostics). Kinetic enzymatic test was performed to evaluate urea, and creatinine was assessed by kinetic staining test (Jaffé method) (Cobas 8000 - Roche Diagnostics). All these tests were performed at the hospital laboratory of each centre involved in the study.

# References

1. Callahan BJ, McMurdie PJ, Rosen MJ, Han AW, Johnson AJ, Holmes SP. DADA2: High-resolution sample inference from Illumina amplicon data. Nat Methods 2016;13:581-3. doi:10.1038/nmeth.3869.

2. Seemann T. Prokka: rapid prokaryotic genome annotation. Bioinformatics 2014;30:2068-9. doi:10.1093/bioinformatics/btu153.

3. Page AJ, Cummins CA, Hunt M, Wong VK, Reuter S, Holden MTG, Fookes M, Falush D, Keane JA, Parkhill J. Roary: rapid large-scale prokaryote pan genome analysis. Bioinformatics 2015;31:3691-3. doi:10.1093/bioinformatics/btv421.

4. Liu CM, Aziz M, Kachur S, Hsueh P-R, Huang Y-T, Keim P, Price LB. BactQuant: An enhanced broad-coverage bacterial quantitative real-time PCR assay. BMC Microbiology 2012;12:56. doi:10.1186/1471-2180-12-56.

5. Gargari G, Taverniti V, Gardana C, Cremon C, Canducci F, Pagano I, Barbaro MR, Bellacosa L, Castellazzi AM, Valsecchi C, et al. Fecal Clostridiales distribution and short-chain fatty acids reflect bowel habits in irritable bowel syndrome. Environ Microbiol 2018;20:3201-13. doi:10.1111/1462-2920.14271.

6. Gargari G, Mantegazza G, Taverniti V, Del Bo C, Bernardi S, Andres-Lacueva C, Gonzalez-Dominguez R, Kroon PA, Winterbone MS, Cherubini A, et al. Bacterial DNAemia is associated with serum zonulin levels in older subjects. Scientific reports 2021;11:11054. doi:10.1038/s41598-021-90476-0.

# SUPPLEMENTARY RESULTS

Figure S1. Receiver Operator Curve (ROC) curve of the PLS discriminant analyses (PLSDA) shown in Figure 3 (panel A) and Sparse PLS discriminant analysis with centroids for sample groups R (responders), NR (non-responders), and HC (healthy controls).


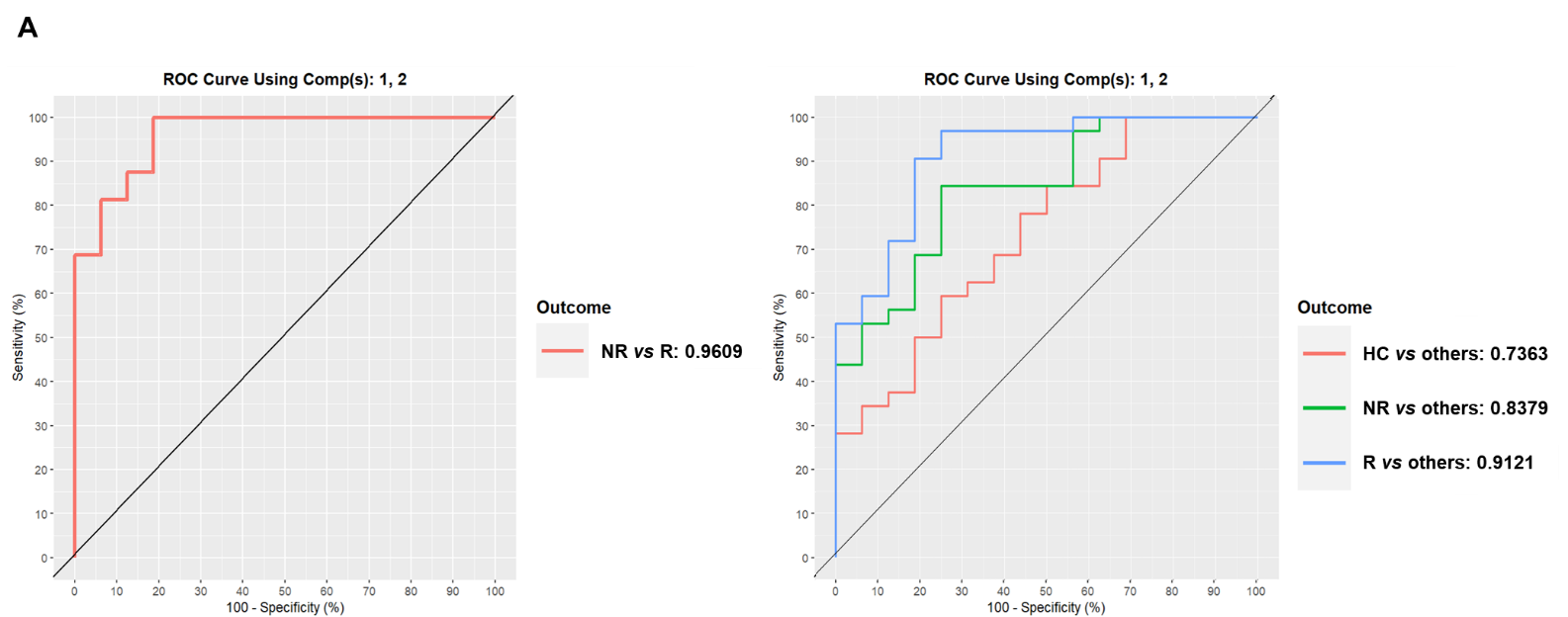


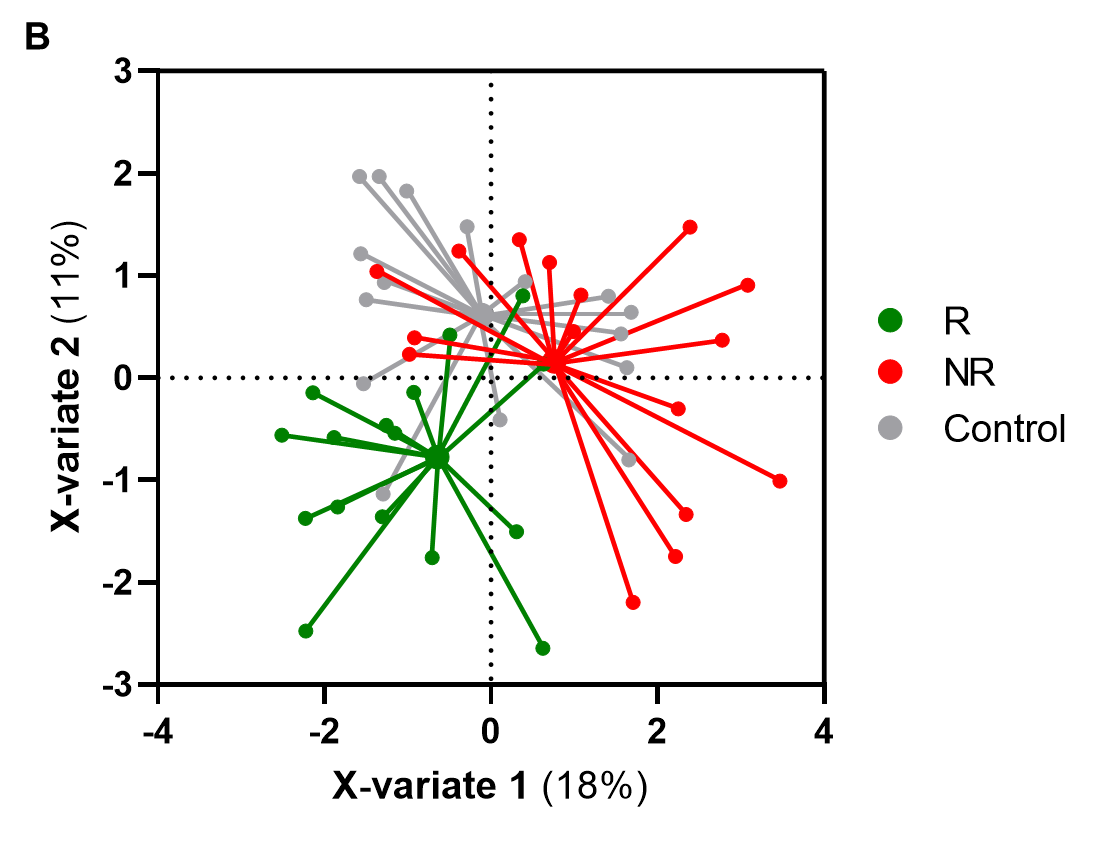


Figure S2. Graphics of Linear discriminant analysis (LDA) effect size (LEfSe) on CLR-transformed taxonomic abundances for all (n=49; **panel A**) and non-responder (n=38; **panel B**) nonconstipated IBS patients in the probiotic arm of the PROBE-IBS/2 trial. LDA scores indicate taxa significantly (P<0.05) higher before (V2; negative LDA) or after (V4; positive LDA) the probiotic intake. The name of the taxon levels is abbreviated as follows: p, phylum; c, class; o, order; f, family; g, genus; s, species. Corrections/updates of the taxonomy with respect to the nomenclature in the GreenGenes database are indicated in violet. Taxonomic names written in blue were determined through a manual BLASTN search in GenBank using the sequence of the corresponding reads. Taxon cAVS 254 was recognized as the probiotic strain administered in this study (*L. paracasei* DG; in green).

**A**


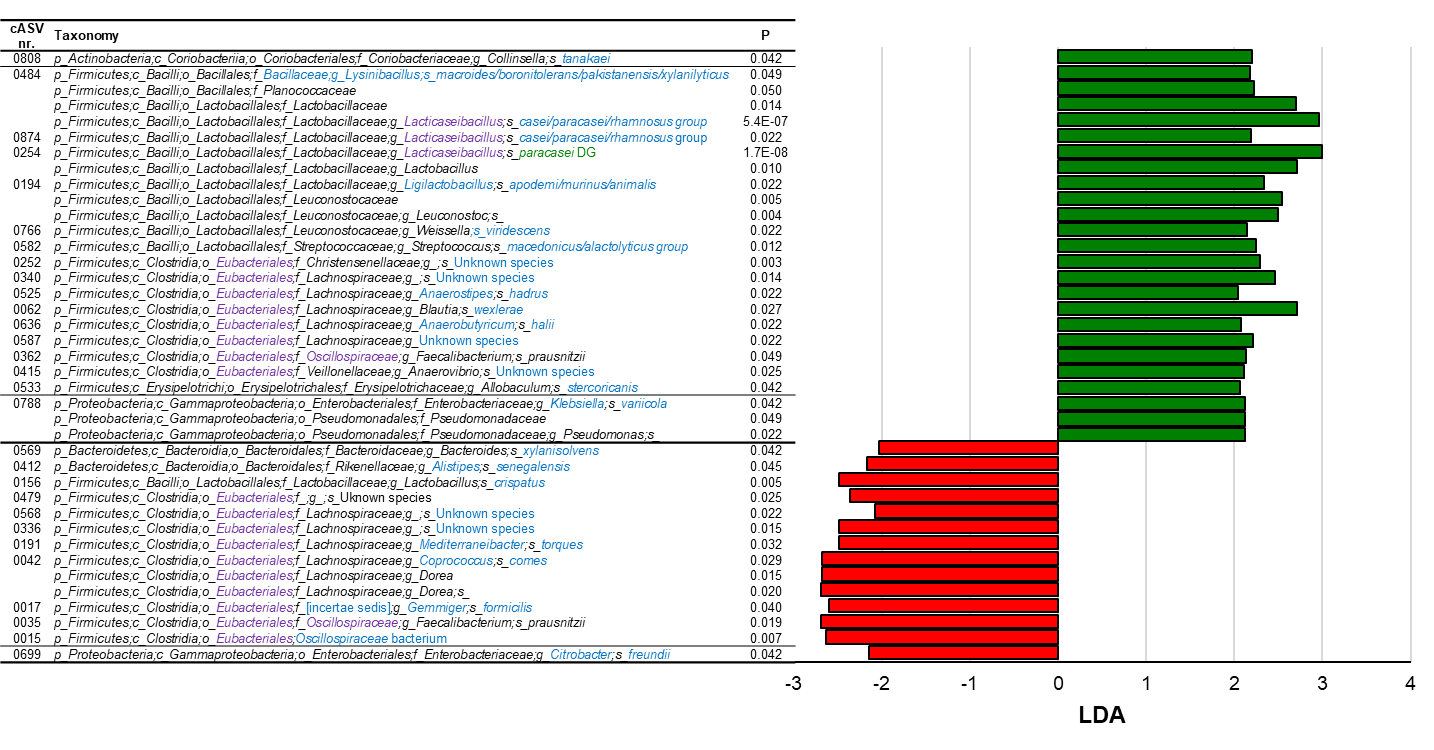


**B**


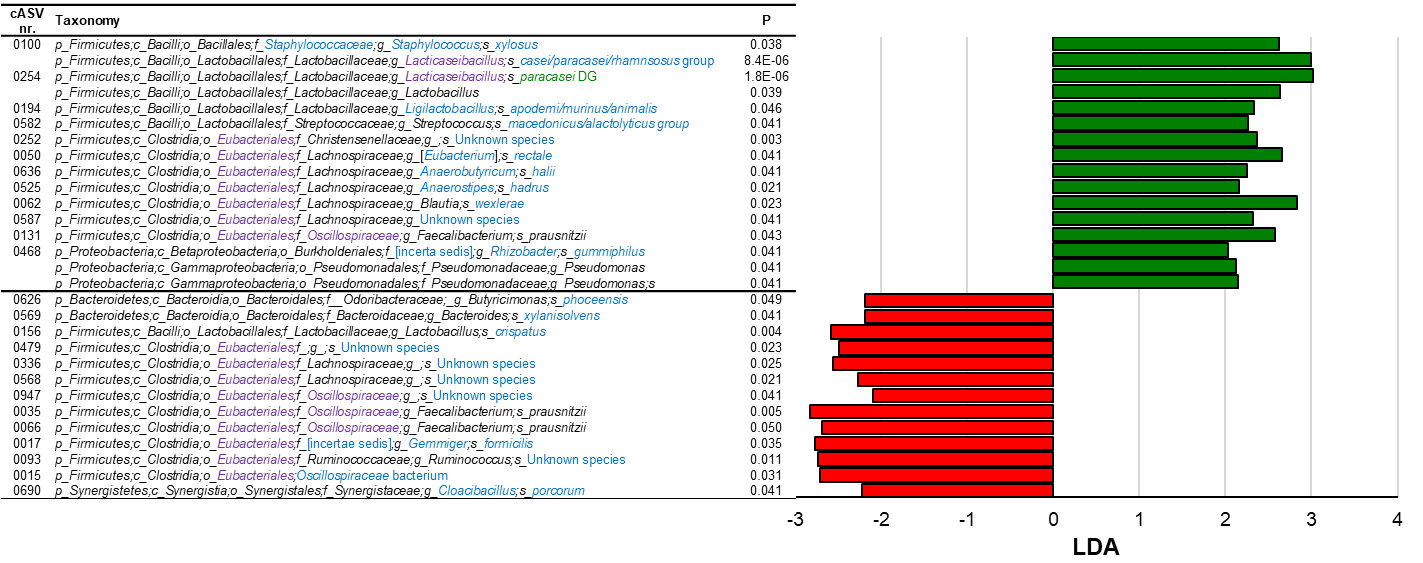


Figure S3. Quantification of the species *Collinsella aerofaciens* via TaqMan real-time quantitative PCR. NR, non-responders; R, responders. The red line indicates the median. +, mean. Statistics is according to Mann-Whitney test (unpaired; NR *vs* R) or Wilcoxon test (paired; V2 *vs* V4).

Figure S4. Correlation analysis between bacterial taxa and serum markers. The heatmap represents the τ coefficient of Kendall rank correlation. Asterisks indicate significance in the Kendall rank correlation: *P < 0.05; **P < 0.01; ***P < 0.001. V1/V2, correlation analysis performed with data from blood samples collected before run-in (visit V1) and fecal samples collected before the probiotic intervention (visit V2); V4, correlation analysis performed with data from blood and fecal samples collected at end of the probiotic intervention (visit V4). The taxonomic lineage of each taxon is shown: p, phylum; c, class; o, order; f, family; g, genus; s, species.

**V4 V1/V2**

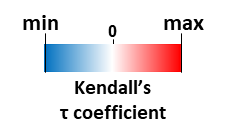

Supplement: Data supplement file_Guglielmetti_R1_CLEAN.docx [file KGMI_A_2298246_SM5056.docx]
